# Cox-2 Inhibition Protects against Hypoxia/Reoxygenation-Induced Cardiomyocyte Apoptosis* via* Akt-Dependent Enhancement of iNOS Expression

**DOI:** 10.1155/2016/3453059

**Published:** 2016-10-04

**Authors:** Lei Pang, Yin Cai, Eva Hoi Ching Tang, Dan Yan, Ramoji Kosuru, Haobo Li, Michael G. Irwin, Haichun Ma, Zhengyuan Xia

**Affiliations:** ^1^Department of Anesthesiology, The First Hospital of Jilin University, Jilin 130021, China; ^2^Department of Anesthesiology, The University of Hong Kong, Pokfulam, Hong Kong; ^3^Department of Pharmacology and Pharmacy and State Key Laboratory of Pharmaceutical Biotechnology, The University of Hong Kong, Pokfulam, Hong Kong; ^4^School of Biomedical Sciences, The University of Hong Kong, Pokfulam, Hong Kong

## Abstract

The present study explored the potential causal link between ischemia-driven cyclooxygenase-2 (COX-2) expression and enhanced apoptosis during myocardial ischemia/reperfusion (I/R) by using H9C2 cardiomyocytes and primary rat cardiomyocytes subjected to hypoxia/reoxygenation (H/R). The results showed that H/R resulted in higher COX-2 expression than that of controls, which was prevented by pretreatment with Helenalin (NF*κ*B specific inhibitor). Furthermore, pretreatment with NS398 (COX-2 specific inhibitor) significantly attenuated H/R-induced cell injury [lower lactate dehydrogenase (LDH) leakage and enhanced cell viability] and apoptosis (higher Bcl2 expression and lower level of cleaved caspases-3 and TUNEL-positive cells) in cardiomyocytes. The amelioration of posthypoxic apoptotic cell death was paralleled by significant attenuation of H/R-induced increases in proinflammatory cytokines [interleukin 6 (IL6) and tumor necrosis factor (TNF*α*)] and reactive oxygen species (ROS) production and by higher protein expression of phosphorylated Akt and inducible nitric oxide synthase (iNOS) and enhanced nitric oxide production. Moreover, the application of LY294002 (Akt-specific inhibitor) or 1400W (iNOS-selective inhibitor) cancelled the cellular protective effects of NS398. Findings from the current study suggest that activation of NF*κ*B during cardiomyocyte H/R induces the expression of COX-2 and that higher COX-2 expression during H/R exacerbates cardiomyocyte H/R injury* via* mechanisms that involve cross talks among inflammation, ROS, and Akt/iNOS/NO signaling.

## 1. Introduction

Ischemic heart disease caused by partial or complete blockage of coronary arteries is a leading cause for morbidity and mortality worldwide, especially in developed countries/regions [1]. It is well known that the most effective intervention to reduce myocardial ischemic injury is restoration of the coronary blood flow. Clinically, restoration of the coronary blood flow improved the survival rate of the patient in general, but the process of myocardial reperfusion itself may also induce additional cardiac damage and complications, referred to as “myocardial ischemia/reperfusion (I/R) injury” [1, 2]. Although the molecular mechanisms mediating reperfusion injury are largely unknown, the underlying pathophysiology of myocardial I/R injury likely involves many factors, such as reactive oxygen species (ROS) formation, activation of cell apoptosis, and inflammatory responses [2, 3].

Cyclooxygenase- (COX-) 1 and COX-2 are the two isoforms of cyclooxygenase, both of which catalyze the transformation of arachidonic acid to prostanoids [4–6]. COX-1 is widely and constitutively distributed in the body while COX-2 is expressed at low levels and is strongly induced in response to different stimuli [7]. Accumulating evidence has proved that COX-2 is highly expressed in the cardiac tissue during myocardial ischemia, suggesting that induction of COX-2 may be involved in ischemic heart disease [8, 9]. Although COX-2 expression after ischemic preconditioning in animal models of myocardial I/R has been considered to be cardioprotective [10, 11], more reports have suggested that enhancement of COX-2 expression plays a detrimental role in the development of myocardial I/R injury [12–15]. In particular, treatment with a COX-2 specific inhibitor significantly improved cardiac function and reduced infarct size after myocardial infarction in different experiment animal models, including rat [12, 13, 16], rabbit [14], and dog [15]. However, it has not been explored how inhibition of COX-2 protects against cardiac damage during myocardial I/R injury.

Necrosis and apoptosis have been considered as major morphologically distinct pathways that regulate tissue damage [17]. For a long time necrosis was believed to be the exclusive cause of cell death during myocardial I/R injury. More recent studies have demonstrated that cardiomyocyte apoptosis also contributes to the process of cardiac damage during I/R injury, which causes the loss of cardiomyocyte volume and the subsequent cardiac dysfunction [18, 19]. Therefore, exploring ways to control or attenuate I/R-induced increase in cardiomyocytes apoptosis is of clinical interest for improving postischemic recovery. A previous study reported that the presence and the content of COX-2 were positively correlated with the severity of apoptosis at the site of acute myocardial infarction [20]. However, whether there is a causal link between ischemia-driven COX-2 expression and apoptosis during I/R injury and in particular the impact of ischemia-induced increases in COX-2 on conventional cardiac prosurvival signaling molecules such as Akt and nitric oxide (NO) [21–23] have not been explored. Thus, the present study investigated the molecular basis of potential cause-effect link between COX-2 expression and apoptosis during ischemia using rat origin cardiomyocytes (H9C2) and primary adult rat cardiomyocytes subjected to hypoxia/reoxygenation (H/R).

## 2. Materials and Methods

### 2.1. H9C2 Cells Culture and Isolation of Primary Adult Rat Cardiomyocyte

The rat cardiomyocyte-derived cell line H9C2 was purchased from the American Type Culture Collection (ATCC, Manassas, VA, USA). H9C2 cells from passage 5 to passage 20 were used in this study. These cells were cultured in Dulbecco's Modified Eagle's Medium (DMEM, Cat. number 11185-084, ThermoFisher Scientific, Waltham, MA, USA) supplemented with 10% fetal bovine serum (FBS, Cat. number FB-1001/500, Biosera, Kansas City, MO, USA) and 1% penicillin/streptomycin (100 U/mL, Cat. number 15140122, ThermoFisher Scientific). Primary adult rat cardiomyocytes were isolated as described [24]. Briefly, hearts from male Sprague-Dawley rat (8 to 10 weeks of age) were rapidly removed and retrogradely perfused at 37°C for 20 min by perfusate including collagenase II (2 mg/mL, Cat. number 17101-015, ThermoFisher Scientific) and CaCl_2_ (50 *μ*M). Subsequently the heart was quickly removed and gently teased into small pieces, and the dispersed myocytes were then filtered through 70 *μ*m nylon cell strainer. The cardiomyocytes were resuspended with M199 medium (Cat. number 11150-059, ThermoFisher Scientific) supplemented with 10% FBS and 1% insulin/transferring/selenium (ITS, Cat. number 13146, Sigma-Aldrich, St. Louis, MO, USA) in 6-well plates precoated with Matrigel (Cat. number 354248, Corning Life Sciences, Corning, NY, USA). All cells were incubated at 37°C in a room air atmosphere containing 5% CO_2_-95% O_2_.

### 2.2. Cell Treatment

The rat cardiomyocytes were randomly divided into three groups: (1) control (CTL) group: the cells were incubated in DMEM medium (5.5 mM glucose, H9C2 cells) or M199 medium (primary rat cardiomyocytes), (2) hypoxia/reoxygenation (H/R) group: since the H9C2 cardiomyocytes and primary adult rat cardiomyocytes showed different sensitivity to the hypoxia and reoxygenation treatment (unpublished observations), then these two kinds of cells were incubated in DMEM medium with no glucose or serum with different duration of time [6 hours (h) (H9C2 cells) or 45 minutes (min) (primary rat cardiomyocytes) for hypoxia and 12 h (H9C2 cells) or 2 h (primary rat cardiomyocytes) for reoxygenation]. Hypoxia was achieved by placing the cells on a humidified Plexiglas chamber, containing 95% N_2_ and 5% CO_2_ to mimic ischemia. Reoxygenation was achieved by exposing cell to normal medium and room air with atmosphere containing 5% CO_2_-95% O_2_ at 37°C, and (3) H/R + Helenalin or H/R + NS398 group: according to the earlier work in the laboratory or from the literature [25, 26], the usage of Helenalin (NF*κ*B specific inhibitor, Cat. number ALX-350-120-MC05, Enzo Life Sciences, Syosset, NY, USA) and NS398 (COX-2 specific inhibitor, Cat. number N194, Sigma) at the following concentration and exposure time exerted significant inhibitory effects on NF*κ*B and COX-2 activity, respectively. The cells were preincubated with Helenalin (10 *μ*M, 2 h) [25] or NS398 (10 *μ*M, 1 h) [26] and followed by H/R treatment. Helenalin or NS398 was added to the changed medium during H/R stimulation to keep the working concentration. At harvest time, cultured medium was snap-frozen in liquid N_2_ and cells pellets were homogenized in TRIzol (Invitrogen Life Technologies, Carlsbad, CA, USA) for RNA extraction or 1x lysis buffer (Cat. number 9803S, Cell Signaling, USA) supplemented with Protease Inhibitor Cocktail (Cat. number P8340, Sigma) for protein extraction for future analysis.

### 2.3. Analysis of LDH Leakage and Cell Viability

Cell injury was evaluated by lactate dehydrogenase (LDH) leakage. The released LDH in the collected medium was determined by using a commercial LDH kit (Cat. number 11644793001 Roche, Mannheim, Germany) according to the manufacturer's instructions. The cell viability of the H9C2 cells and primary adult rat cardiomyocytes was determined by the 3-(4,5-dimethylthiazol-2-yl)-2,5-diphenyltetrazolium bromide (MTT) assay as described [27, 28]. Briefly, cells were seeded in 96-well plates and were incubated with MTT solution (1 mg/mL, Cat. number M5655, Sigma) at 37 °C for 4 h after the various treatments. The formazan crystals were dissolved with dimethyl sulfoxide (DMSO, 100 mL/well), and the absorbance was detected at 570 nm with Epoch microplate spectrophotometer (BioTek, Winooski, VT, USA).

### 2.4. Real-Time Polymerase Chain Reaction

Total RNA was extracted from H9C2 cells using TRIzol. Equal amounts of RNA were reverse-transcribed and processed using the PrimeScript RT Master Mix Kit (Cat. number RR036Q, Takara, Shuzou, Japan), according to the manufacturer's instructions. Quantitative real-time PCR was performed as described [29] using a SYBR Green PCT master mix (Cat. number RR820A, Takara) on an Applied Biosystems Prism 7000 sequence detection system (Applied Biosystems, Foster City, CA, USA). The conditions for amplification were 30 seconds at 95°C for denaturation, 40 cycles of 5 seconds at 95°C, and 30 seconds at 60°C. The gene-specific primers sequences used were listed in Table 1. The mRNA levels of the different genes tested were normalized to those of *β*-actin.

### 2.5. Western Blotting

Total cell lysates were prepared by lysing primary adult rat cardiomyocytes or H9C2 cells with 1x lysis buffer supplemented with Protease Inhibitor Cocktail. The protein concentration of the samples was determined with the Bradford assay (Cat. number 500-0006, Bio-Rad, Hercules, CA, USA). Equal amounts of protein from each sample were separated on 8%–12.5% sodium dodecyl sulfate-polyacrylamide gel electrophoresis and transferred onto PVDF membranes for detection with appropriate antibodies. Primary antibodies against COX-2 (1 : 1000, Cat. number 160106) and inducible nitric oxide synthase (iNOS, 1 : 40, Cat. number ab15323) were purchased from Cayman (Ann Arbor, MI, USA) and Abcam (Cambridge, MA, USA), respectively. Primary antibodies against Bcl2-like protein 4 (Bax, 1 : 1000, Cat. number 2772S), Bcl2 (1 : 1000, Cat. number 2870S), glyceraldehyde 3-phosphate dehydrogenase (GADPH, 1 : 1000, Cat. number 5174S), inhibitor of kappa B *α* (I*κ*B*α*, 1 : 1000, Cat. number 4814S), phospho-I*κ*B*α* (1 : 1000, Cat. number 9246S), p65 (1 : 1000, Cat. number 4764S), phospho-p65 (1 : 1000, Cat. number 3033S), total caspase-3 (1 : 1000, Cat. number 9665S), cleaved caspase-3 (1 : 1000, Cat. number 9661S), total Akt (1 : 1000, Cat. number 9272S), phosphor-Akt (1 : 1000, T450 Cat. number 9267S), and horseradish peroxidase-conjugated anti-mouse (Cat. number 7076S) or anti-rabbit (Cat. number 7074S) secondary antibodies (1 : 3000) were purchased from Cell Signaling Technology (Danvers, MA, USA). Blots were visualized with Clarity Western Blotting Detection Reagent (Cat. number 102030726, Bio-Rad) and subsequently exposed to X-ray film (Carestream, NY, USA). ImageJ software (National Institutes of Health, MD, USA) was used to analyze the optical densities of the immunoreactive bands as described [30].

### 2.6. TUNEL Assay

H9C2 cardiomyocytes were cultured directly on coverslips. After the treatment, the cardiomyocytes' apoptosis was determined by TUNEL staining using an In Situ Cell Death Detection Kit (Cat. number 11684817910, Roche Diagnostics GmbH, Mannheim, Germany), according to the manufacturer's instructions. Images were taken using an Olympus BX41 fluorescence microscope equipped with an Olympus DP72 color digital camera (Olympus, Tokyo, Japan). Five photos (magnification ×200) were taken randomly for each sample. Image quantification was presented as percentage of TUNEL-positive cells among the total number of cells as described [31].

### 2.7. Determination of Cyclooxygenase-2 Activity

The COX-2 activity in H9C2 cell lysates from different groups (CTL, H/R, and H/R + NS398) was determined by a commercial COX Fluorescent Activity Assay Kit (Cat. number 700200, Cayman). The COX-2 activity was detected in the presence of SC560 (5 *μ*M, COX-1 selective inhibitor) according to the manufacturer's instructions.

### 2.8. Measurement of Intracellular Reactive Oxygen Species and Nitric Oxide Production

The H9C2 cells were seeded in 96-well black polystyrene plates (Cat. number 3916, Corning Life Sciences) at subconfluent level in the presence of NS398 (10 *μ*M for 1 h) or LY294002 (10 *μ*M for 1 h) [32] or 1400W (1 *μ*M for 30 mins) [33] with or without H/R stimulation. To detect the intracellular level of reactive oxygen species (ROS), the cells were incubated with dihydroethidium fluorescence (DHE, 8 *μ*M, Cat. number 37291, Sigma) in a Krebs-HEPES buffer at 37°C in dark for 30 min. After cells were washed, fluorescence was measured in a CLARIOstar Monochromator Microplate Reader (BMG LABTECH, Ortenberg, Germany) by using 518 and 605 nm wavelength excitation and emission filters, respectively. To detect the intracellular nitric oxide (NO) production, 4-amino-5-methylamino-2′,7′-difluorofluorescein diacetate (DAF-FM diacetate, Cat. number D-23844, ThermoFisher Scientific, Waltham, MA, US), which becomes fluorescent when it is nitrosylated by oxidation products of NO, was added to the H9C2 cells in a working concentration of 10 *μ*M and allowed to incubate at 37°C in dark for 30 min. After cells were washed, fluorescence was detected in a CLARIOstar Monochromator Microplate Reader with 495 nm excitation and 515 nm emission.

### 2.9. Statistical Analysis

All data are expressed as the means ± SEM. Comparison between two groups was carried out by two-tailed nonparametric Mann–Whitney* U* test or one-way ANOVA followed by Tukey's test, wherever appropriate, using the GraphPad Prism 5.0 software (San Diego, CA, USA). Differences were considered statistically significant when *P* was less than 0.05.

## 3. Results

### 3.1. NF*κ*B Mediates the H/R-Induced Expression of COX-2 in H9C2 Cardiomyocytes

**Figure 1:**
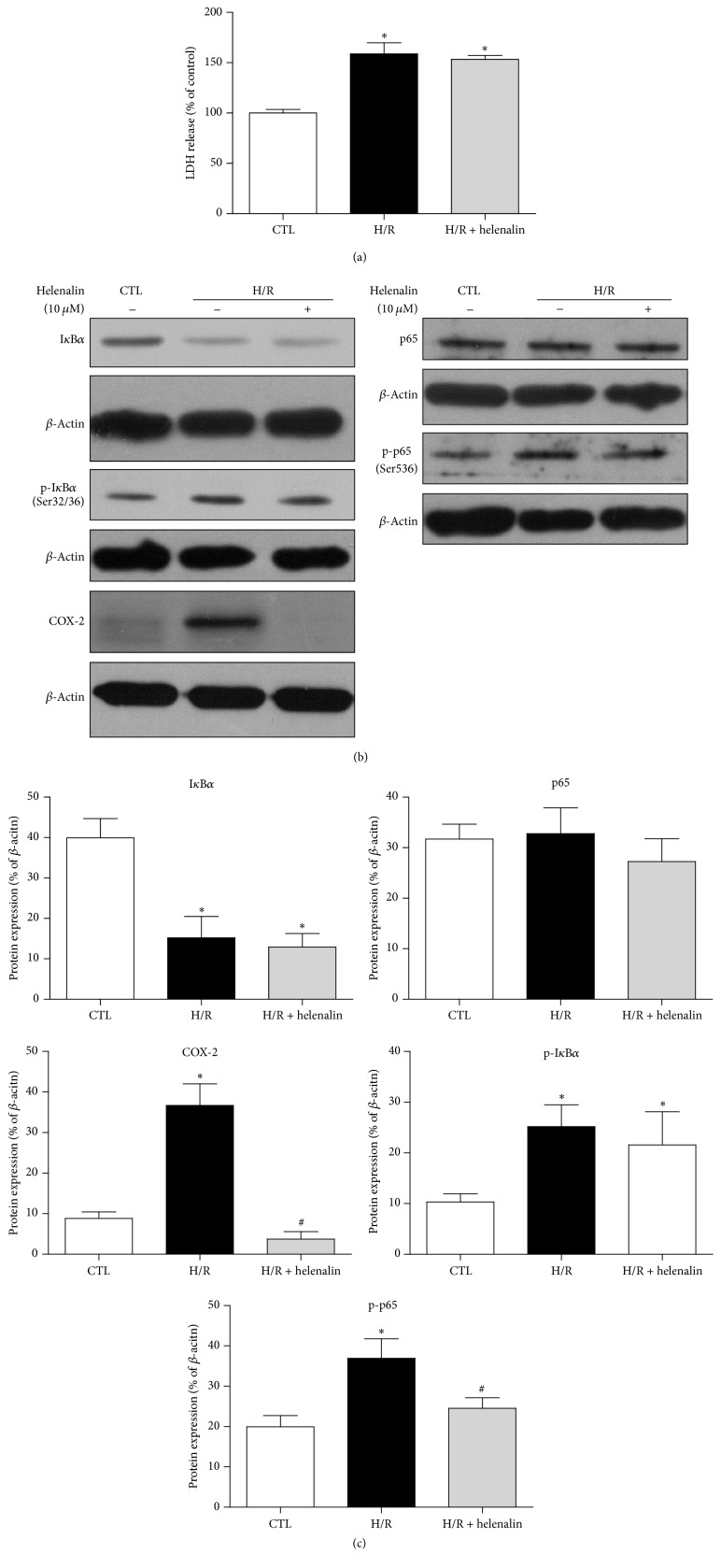
Activation of NF*κ*B mediates the H/R-induced expression of COX-2 in H9C2 cardiomyocytes. (a) The release of LDH (cell injury marker) and (b) representative original Western blots of I*κ*B*α*, phosphorylated I*κ*B*α* (S32/36), p65, phosphorylated p65 (S536), and COX-2 in H9C2 cardiomyocytes with or without H/R stimulation (6 h hypoxia followed by 12 h reoxygenation) in the presence or absence of Helenalin (NF*κ*B specific inhibitor, 10 *μ*M, 2 h). (c) Quantification of these proteins after normalization to *β*-actin. Data are shown as means ± SEM; ^*∗*^ *P* < 0.05 CTL versus H/R or H/R + Helenalin, ^#^ *P* < 0.05 H/R versus H/R + Helenalin (one-way ANOVA followed by Tukey's test in (a) and nonparametric Mann–Whitney* U* test in (c)); *n* = 4 per group.

The LDH release was higher after H/R stimulation, suggesting the establishment of H/R-induced injury model in cardiomyocytes (Figure 1(a)). The expression of COX-2 was significantly higher when compared with control group (Figures 1(b) and 1(c)). The induction of COX-2 was accompanied by the activation of NF*κ*B, as evidenced by degradation of I*κ*B*α*, and phosphorylation of I*κ*B*α* and p65 in H/R-treated H9C2 cells (Figures 1(b) and 1(c)). NF*κ*B, when being activated, translocates to the nucleus and initiates target genes transcription, including COX-2 in different kinds of cells [29, 34, 35]. To test whether or not the induction of COX-2 was mediated by NF*κ*B in cardiomyocytes during H/R, the H9C2 cells were pretreated by the selective NF*κ*B inhibitor, Helenalin. Despite the comparable LDH leakage and expressions of I*κ*B*α* and phosphorylated I*κ*B*α*, the expression of phosphorylated p65 was significantly lower in H/R + Helenalin group when compared with H/R group, suggesting that Helenalin inhibited NF*κ*B activity through the inhibition of p65 phosphorylation but not the degradation of I*κ*B*α* (Figures 1(a), 1(b), and 1(c)). As anticipated, pretreatment with Helenalin significantly suppressed COX-2 induction in H9C2 cells exposed to H/R (Figures 1(b) and 1(c)), suggesting that activated NF*κ*B mediated the induction of COX-2 in H/R-treated H9C2 cardiomyocytes.

### 3.2. Inhibition of COX-2 Activity Attenuates H/R-Induced Cell Injury

**Figure 2:**
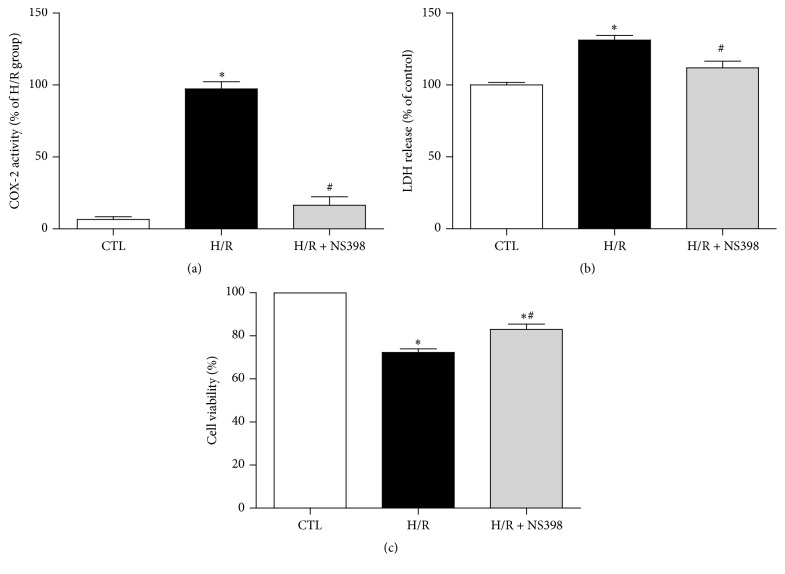
Inhibition of COX-2 activity attenuates H/R-induced cell injury. (a) The COX-2 activity, (b) the release of LDH, and (c) cell viability in H9C2 cardiomyocytes with or without H/R treatment in the presence or absence of NS398 (COX-2 specific inhibitor, 10 *μ*M, 1 h). COX-2 activity was expressed against that in H/R group. The LDH leakage and cell viability were expressed against those in CTL group. Data are shown as means ± SEM; ^*∗*^ *P* < 0.05 CTL versus H/R or H/R + NS398, ^#^ *P* < 0.05 H/R versus H/R + NS398 (one-way ANOVA followed by Tukey's test); *n* = 5 per group.

To observe whether or not the higher expression of COX-2 contributed to H/R-induced cell injury in H9C2 cardiomyocytes, the cells were incubated with the COX-2 selective inhibitor, NS398, before being subjected to H/R. Consistent with the higher COX-2 protein level in response to H/R stimulation, the activity of COX-2 in the H/R group was also significantly higher as compared to control cells, and such augmentation in COX-2 activity was significantly suppressed by NS398 (Figure 2(a)). This suggests that NS398 pretreatment was effective in inhibiting H/R-induced COX-2 activity.

LDH leakage is a biomarker of cell injury [36]. As shown in Figure 2, the release of LDH to the culture medium was significantly higher after H/R stimulation as compared to control cells, whereas this LDH release was significantly inhibited by pretreatment with NS398 when compared with H/R group (Figure 2(b)). Consistently, cell viability was significantly reduced after H/R stimulation, while pretreatment with NS398 significantly enhanced the cell viability when compared with H/R group (Figure 2(c)). Taken together, these observations suggest that inhibition of COX-2 activity significantly attenuated H/R-induced cell injury.

### 3.3. Inhibition of COX-2 Activity Alleviates H/R-Induced Cell Apoptosis in H9C2 Cardiomyocytes

**Figure 3:**
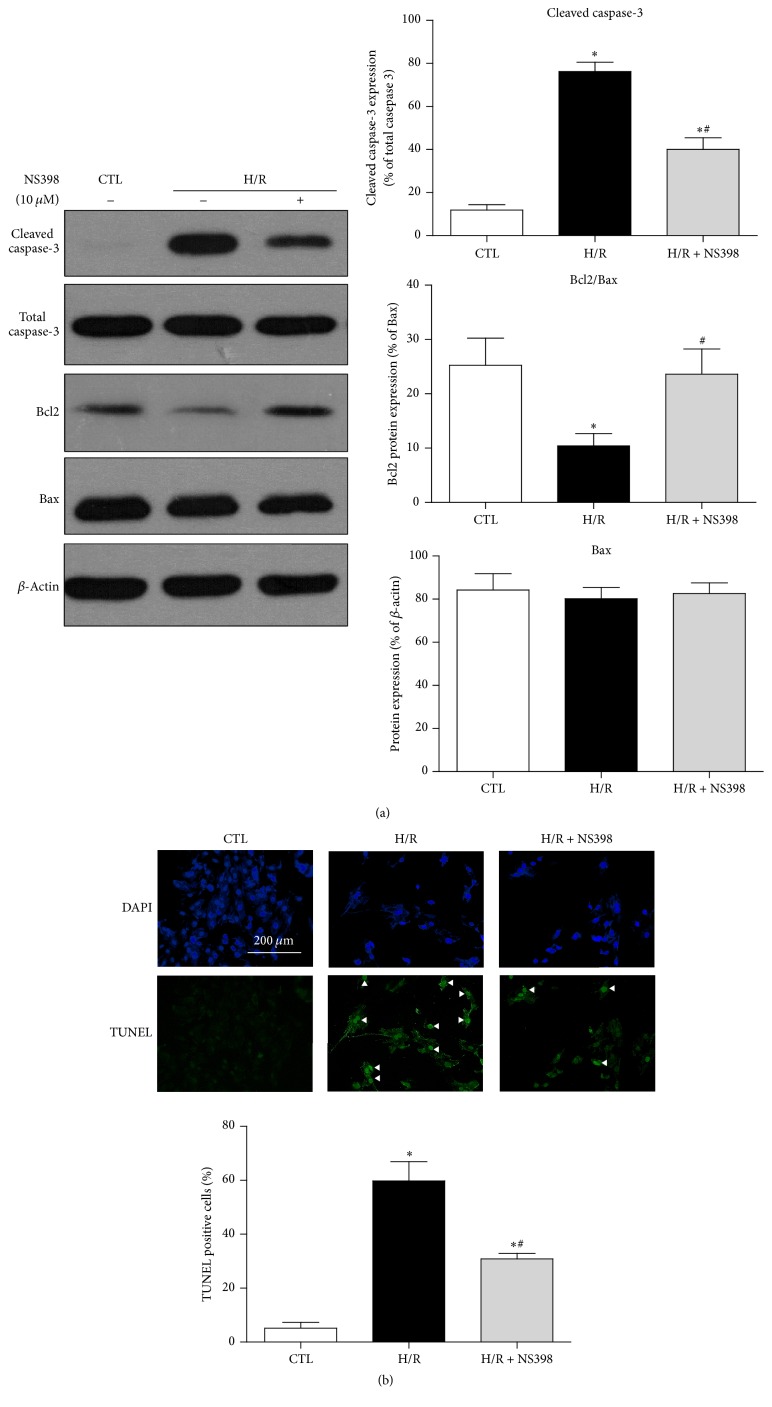
Inhibition of COX-2 activity alleviates H/R-induced cell apoptosis in H9C2 cardiomyocytes. (a) Representative original Western blots of apoptosis related proteins (cleaved caspase-3, total caspase-3, Bcl2, and Bax) in H9C2 cardiomyocytes with or without H/R stimulation in the presence or absence of NS398. Protein presence of cleaved caspase-3 and Bcl2 was normalized to total caspase-3 and Bax, respectively. (b) Representative fluorescence microscopy images of TUNEL staining. White triangle points to positive staining cells (green). DAPI (blue) indicates the nucleus staining. Magnification ×200, scale bars: 100 *μ*m. TUNEL-positive cells% = TUNEL-positive cell numbers/total cell numbers × 100%. Data are shown as means ± SEM; ^*∗*^ *P* < 0.05 CTL versus H/R or H/R + NS398, ^#^ *P* < 0.05 H/R versus H/R + NS398 (nonparametric Mann–Whitney* U* test in (a) and one-way ANOVA followed by Tukey's test in (b)); *n* = 5 per group.

Apoptosis is the hallmark of reperfusion injury in cardiomyocytes, leading to late cell death [18, 19]. Thus, it is tempting to speculate that pretreatment with NS398 may alleviate H/R-induced cardiomyocytes apoptosis. To test this hypothesis, the levels of apoptotic proteins, including Bax, Bcl2, total caspase-3, and cleaved caspase-3, were determined. The expression of Bax was not affected by H/R stimulation in the current settings of cardiomyocyte H/R model, whereas the expression of Bcl2 was significantly decreased and the expression of cleaved caspase-3 was drastically enhanced in the H/R group as compared to control cells. Pretreatment with NS398 significantly reduced expression of cleaved caspase-3 and enhanced Bcl2 expression (Figure 3(a)), resulting in significant attenuation of H/R induced increases in apoptotic cell death (Figure 3(b)) as measured by TUNEL assay which detects the DNA fragmentation, a characteristic feature of cell apoptosis [37]. Taken together, these data demonstrated that inhibition of COX-2 alleviated H/R-induced cell apoptosis in H9C2 cardiomyocytes.

### 3.4. COX-2 Mediates H/R-Induced Cell Injury and Apoptosis in Primary Adult Rat Cardiomyocytes

**Figure 4:**
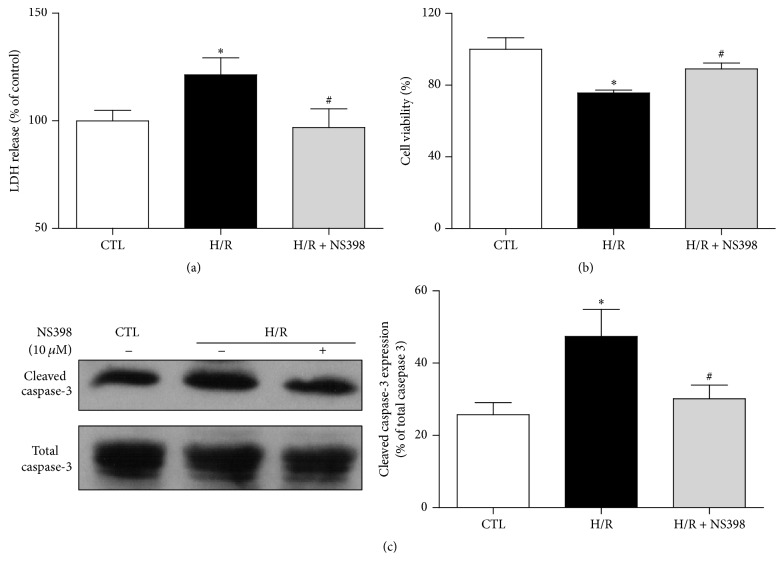
Inhibition of COX-2 activity attenuates H/R-induced cell injury and apoptosis in primary adult rat cardiomyocytes. (a) The release of LDH and (b) cell viability in primary adult rat cardiomyocytes with or without H/R treatment (45 min hypoxia followed by 2 h reoxygenation) in the presence or absence of NS398. The LDH leakage and cell viability were expressed against those in CTL group. (c) Representative original Western blots of cleaved caspase-3 and total caspase-3 in primary adult rat cardiomyocytes with or without H/R treatment in the presence or absence of NS398. Quantification of cleaved caspase-3 was normalized to total caspase-3. Data are shown as means ± SEM; ^*∗*^ *P* < 0.05 CTL versus H/R, ^#^ *P* < 0.05 H/R versus H/R + NS398 (one-way ANOVA followed by Tukey's test in (a), (b) and nonparametric Mann–Whitney* U* test in (c)); *n* = 5 per group.

The present study primarily relied on work done on H9C2 cardiomyocytes to consolidate the effect of COX-2 on H/R-induced cell injury and apoptosis; experiments were repeated on isolated primary adult rat cardiomyocytes. Similar to that observed with H9C2 cardiomyocytes, H/R remarkably augmented the release of LDH and the expression of cleaved caspase-3 and reduced cell viability (Figure 4). Pretreatment with NS398 significantly reduced LDH leakage (Figure 4(a)), improved cell viability (Figure 4(b)), and alleviated expression of cleaved caspase-3 (Figure 4(c)). Taken together, these data illustrated that inhibition of COX-2 also attenuated H/R-induced cell injury and apoptosis in primary rat cardiomyocytes.

### 3.5. Inhibition of COX-2 Activity Reduced H/R-Induced Proinflammatory Cytokines in H9C2 Cardiomyocytes

**Figure 5:**
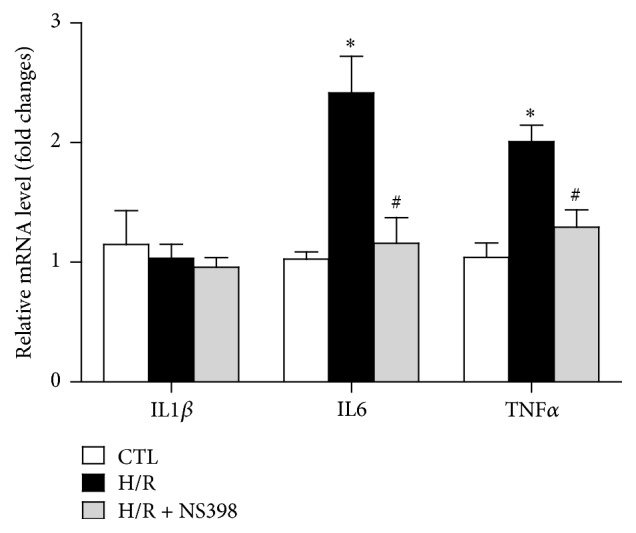
Inhibition of COX-2 activity reduced H/R-induced proinflammatory cytokines expression in H9C2 cardiomyocytes. mRNA expression of proinflammatory genes (IL1*β*, IL6, and TNF*α*) in H9C2 cardiomyocytes subjected to H/R stimulation in the presence or absence of NS398. mRNA levels are expressed against those in H9C2 cardiomyocytes with no stimulation. Data are shown as means ± SEM; ^*∗*^ *P* < 0.05 CTL versus H/R or H/R + NS398, ^#^ *P* < 0.05 H/R versus H/R + NS398 (one-way ANOVA followed by Tukey's test); *n* = 6 per group.

Inducible COX-2 predominantly accounted for the formation of prostanoids associated with inflammation [38]. Thus, the present study investigated whether or not inhibition of COX-2 affects the mRNA expression of H/R-induced proinflammatory cytokines in H9C2 cardiomyocytes. As shown in Figure 5, H/R stimulation selectively induced the mRNA expression of IL6 and TNF*α* but not IL1*β* mRNA. Such higher expression of IL6 and TNF*α* was significantly suppressed with the NS398 pretreatment, suggesting that COX-2 may favor proinflammatory environment in H/R-stimulated H9C2 cardiomyocytes.

### 3.6. Inhibition of COX-2 Activity Reduced H/R-Induced Intracellular ROS Level in H9C2 Cardiomyocytes

**Figure 6:**
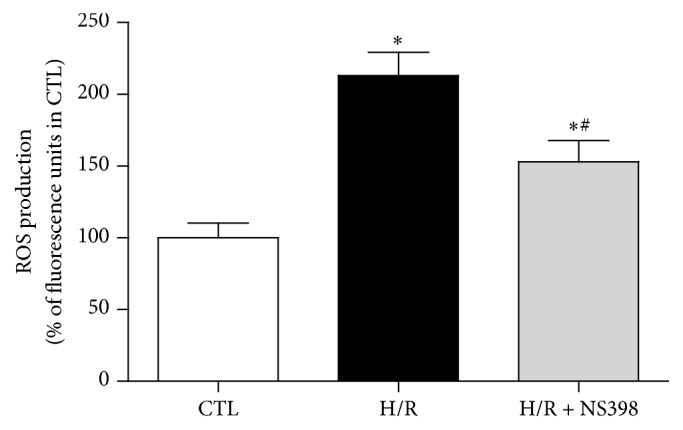
Inhibition of COX-2 activity reduced H/R-induced intracellular ROS level in H9C2 cardiomyocytes. ROS production was detected in H9C2 cardiomyocytes with or without H/R stimulation in the presence or absence of NS398 and it is expressed against that in CTL group. Data are shown as means ± SEM; ^*∗*^ *P* < 0.05 CTL versus H/R or H/R + NS398, ^#^ *P* < 0.05 H/R versus H/R + NS398 (one-way ANOVA followed by Tukey's test); *n* = 6 per group.

Generation of reactive oxygen species (ROS) increases during myocardial I/R and it plays a central role in the pathogenesis of myocardial I/R injury [39]. Given that ROS are formed as byproducts during the synthesis of prostanoids by COX [4], hence the present study tested whether or not inhibition of inducible COX-2 may block the H/R-induced intracellular ROS production in H9C2 cardiomyocytes. As shown in Figure 6, the ROS level was significantly higher in H/R group when compared to control H9C2 cardiomyocytes. However, such H/R-induced increase in ROS production was significantly suppressed by NS398 pretreatment, suggesting that inhibition of COX-2 activity may protect against H/R-induced cell injury and apoptosis through attenuation of ROS production.

### 3.7. Inhibition of COX-2 Activates Akt/iNOS/NO Signaling Pathway in H9C2 Cardiomyocytes

**Figure 7:**
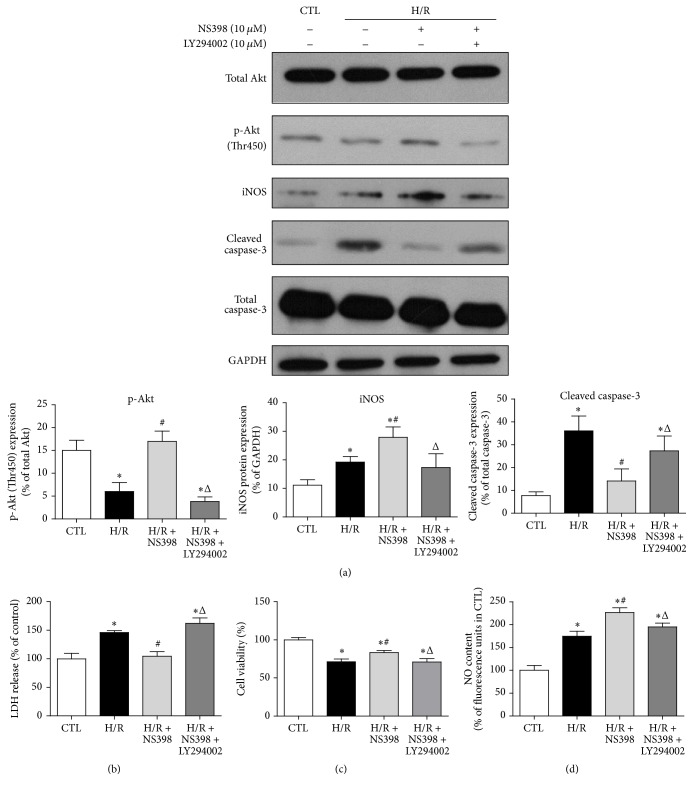
Inhibition of COX-2 activity protects against H/R-induced cell apoptosis* via* Akt-dependent enhancement of iNOS expression. (a) Representative original Western blots of total Akt, phosphorylated Akt (T450), iNOS, cleaved caspase-3, and total caspase-3 in H9C2 cardiomyocytes with or without H/R stimulation in the presence or absence of NS398 or LY294002 (Akt-specific inhibitor, 10 *μ*M, 1 h). Phosphorylated Akt, iNOS, and cleaved caspase-3 were normalized to total Akt, GAPDH, and total caspase-3, respectively. (b) The release of LDH, (c) cell viability, and (d) NO content were determined in H9C2 cardiomyocytes with or without H/R stimulation in the presence or absence of NS398 or LY294002 and were expressed against those in CTL group. Data are shown as means ± SEM; ^*∗*^ *P* < 0.05 CTL versus H/R or H/R + NS398 or H/R + NS398 + LY294002, ^#^ *P* < 0.05 H/R versus H/R + NS398, and ^Δ^ *P* < 0.05 H/R + NS398 versus H/R + NS398 + LY294002 (nonparametric Mann–Whitney* U* test in (a) and one-way ANOVA followed by Tukey's test in (b), (c), and (d)); *n* = 5 per group.

The Akt signaling cascade plays an important role in controlling cardiomyocytes survival and apoptosis [40]. To investigate whether or not NS398 pretreatment may affect the Akt pathway, H9C2 cardiomyocytes were subjected to H/R in the absence or presence of NS398 and total Akt and phosphorylated Akt protein (T450) expression was assessed. H/R significantly reduced the expression of phosphorylated Akt, but not total Akt level (Figure 7(a)). The level of phosphorylated Akt in H/R-stimulated H9C2 cells was significantly higher by NS398 pretreatment, suggesting that inhibition of COX-2 may activate Akt pathway and thus exert a cardioprotective effect (Figure 7(a)). To confirm our speculation, LY294002, the specific Akt inhibitor, was applied in the present study. As anticipated, the NS398-induced augmentation of phosphorylated Akt was significantly suppressed by LY294002 pretreatment (Figure 7(a)). In addition, NS398 mediated reduction of LDH leakage and cleaved caspase-3 level and increase of cell viability was abolished in the presence of LY294002 (Figures 7(a), 7(b), and 7(c)). This suggests that activation of Akt mediated the cardioprotective effect of NS398.

**Figure 8:**
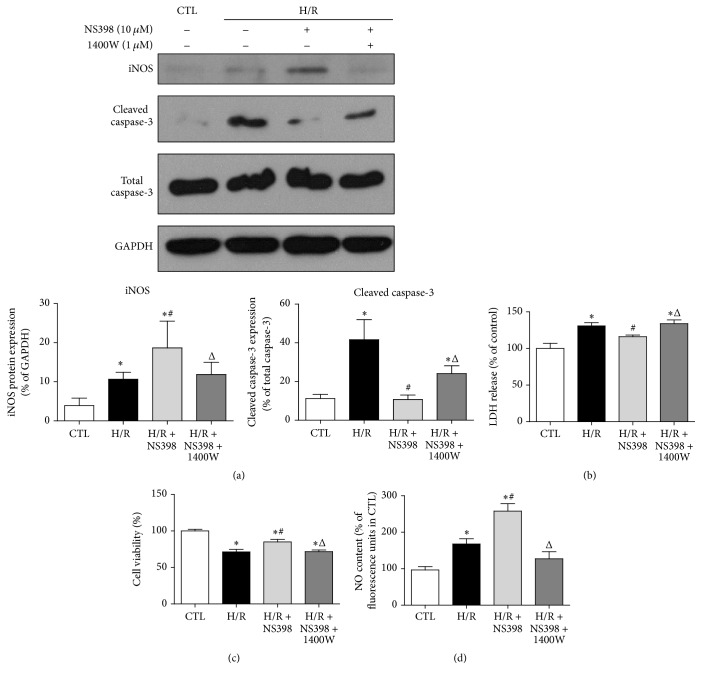
iNOS primarily mediated cardioprotective effects of NS398 in H/R-induced H9C2 cardiomyocytes apoptosis. (a) Representative original Western blots of iNOS, cleaved caspase-3, total caspase-3, and GAPDH in H9C2 cardiomyocytes with or without H/R stimulation in the presence or absence of NS398 or 1400W (iNOS specific inhibitor, 1 *μ*M, 30 min). iNOS and cleaved caspase-3 were normalized to GAPDH and total caspase-3, respectively. (b) The release of LDH, (c) cell viability, and (d) NO content were determined in H9C2 cardiomyocytes with or without H/R stimulation in the presence or absence of NS398 or 1400W and were expressed against those in CTL group. Data are shown as means ± SEM; ^*∗*^ *P* < 0.05 CTL versus H/R or H/R + NS398 or H/R + NS398 + 1400W, ^#^ *P* < 0.05 H/R versus H/R + NS398, and ^Δ^ *P* < 0.05 H/R + NS398 versus H/R + NS398 + 1400W (nonparametric Mann–Whitney* U* test in (a) and one-way ANOVA followed by Tukey's test in (b), (c), and (d)); *n* = 5 per group.

iNOS is the known downstream target of activated Akt [41] and moderate increase of iNOS has been shown to be cardioprotective during myocardial I/R injury through the synthesis of NO [42]. It is feasible to hypothesize that NS398 may protect against H/R-induced cell apoptosis through Akt/iNOS pathway in H9C2 cardiomyocytes. Indeed, the expression of iNOS was significantly higher by NS398 pretreatment as compared to H/R group (Figure 7(a)). However, such NS398-mediated increase in iNOS expression was blocked by LY294002, suggesting that iNOS worked downstream of Akt in the current settings of cardiomyocyte H/R (Figure 7(a)). To determine whether or not the altered iNOS protein expression is related to intracellular NO production, the NO level was compared among various groups. As anticipated, pretreatment with NS398 resulted in higher NO production as compared to H/R group, whereas LY294002 significantly downregulated the NS398-mediated increase in intracellular NO production (Figure 7(d)). To further confirm whether or not iNOS was the primary mediator through which NS398 mediated cardioprotective effects, the iNOS-selective inhibitor 1400W was applied in the present study. As expected, the NS398-induced augmentation of iNOS expression was significantly suppressed in the presence of 1400W (Figure 8(a)). In addition, the reduction of cleaved caspase-3 level and LDH leakage and higher cell viability in the H/R + NS398 group was almost diminished in the presence of 1400W (Figures 8(a), 8(b), and 8(c)). Furthermore, the NS398-mediated increase in intracellular NO production was significantly downregulated by 1400W pretreatment (Figure 8(d)). Taken together, these observations suggest that inhibition of COX-2 activity protected against H/R-induced cell apoptosis through activation of Akt/iNOS/NO pathway.

## 4. Discussion

COX-2 is expressed at very low level at physiological condition but is strongly induced in response to different stimuli, including proinflammatory cytokines, hypoxia, and mechanical stress [9, 34, 43]. The present study demonstrated that COX-2 expression was significantly higher in H9C2 cardiomyocytes in response to H/R stimulation, which was correspondent to severe posthypoxic cardiomyocyte injury. This suggests that enhancement of COX-2 may be a mechanism of myocardial I/R injury. Our findings are in line with several previous studies which showed that treatment with COX-2 selective inhibitor significantly improved cardiac function and reduced myocardial infarction size in different myocardial I/R animal models [12–15]. However, up to now, no study has been conducted to address how inhibition of COX-2 activity couples to multiple signaling molecules and exerts cardioprotective effects. In the present study, COX-2 inhibition with NS398 significantly enhanced cell viability and attenuated H/R-induced LDH release, the expression of cleaved caspase-3, and TUNEL-positive cell numbers in cardiomyocytes (H9C2 cells and primary rat cardiomyocytes). This demonstrated that inhibition of COX-2 activity attenuated H/R-induced cell injury and apoptosis. Furthermore, NS398 pretreatment significantly inhibited proinflammatory cytokine expressions (IL6 and TNF*α*), reduced the formation of ROS, and enhanced the activation of Akt/iNOS/NO signaling pathway, which could represent the mechanisms whereby COX-2 inhibition attenuated H/R-induced cell apoptosis in cardiomyocytes.

There are two main mechanisms, intrinsic and extrinsic pathways, that induce the apoptotic cascade [44]. In the intrinsic pathway, in response to stress signal, proapoptotic proteins (such as Bax) disrupt the mitochondria membrane integrity and cause mitochondrial dysfunction and the release of Cytochrome C [44, 45], while the antiapoptotic proteins (such as Bcl2, Bcl2-like protein 2) prevent cell apoptosis by interfering with proapoptotic member aggregation [46, 47]. The released Cytochrome C forms a complex, named apoptosome in the cytoplasm with ATP and Apaf-1, which in turn activates caspase-9 and its downstream caspase-3, the effector protein that initiates DNA fragmentation and the degradation of cytoskeletal and nuclear proteins [48]. In the extrinsic pathway, upon ligand binding to death receptors (such as TNF receptor) on the cell surface, the death-inducing signaling complex is formed and activates caspase-8 and caspase-10, which can cleave caspase-3 and converge the extrinsic and intrinsic caspase cascades [16, 44]. Previous findings indicated that H/R stimulation induced the cardiomyocytes apoptosis using both the intrinsic and extrinsic pathways [16, 49, 50], whereas different duration of H/R stimulation may initiate different apoptotic pathway in cardiomyocytes. In the present study, H/R stimulation should have induced cell apoptosis using both the intrinsic and extrinsic ways, as evidenced by lower expression of Bcl-2/Bax ratio and enhanced cleaved caspase-3 level and TUNEL-positive cell numbers in the H/R group relative to that in the control group H9C2 cardiomyocytes. Of note, there was no change in the protein expression of Bax upon H/R stimulation, which could be due to the specific H/R condition (6 h hypoxia and 12 h reoxygenation) in the current experiment setting. The fact that pretreatment with NS398 can significantly reduce the above-mentioned detrimental effects of H/R stimulation and enhance posthypoxic H9C2 cardiomyocyte viability indicates that inhibition of COX-2 activity may protect against H/R-induced cell injury through attenuation of cell apoptosis.

The next challenge was to examine how inhibition of COX-2 protects against H/R-induced cell apoptosis. During myocardial I/R injury, sustained proinflammatory environment (infiltration of inflammatory cells, release of proinflammatory cytokines and chemokines) favors the cardiomyocytes apoptosis, leading to the loss of cardiomyocyte volume and the subsequent cardiac dysfunction [51]. Given the fact that prostaglandins—by the action of COX—play a key role in the generation of the inflammatory response [38], inhibition of COX-2 activity may attenuate H/R-induced cell apoptosis through blocking the expression of proinflammatory cytokines. Indeed, pretreatment with NS398 significantly reduced H/R-induced mRNA expression of IL6 and TNF*α*, both of which have been well established to possess proapoptotic effects [52, 53]. In particular, through binding with TNF receptor on the cell surface, TNF*α* can initiate extrinsic apoptotic pathway and favor the formation of death-inducing signaling complex, leading to cell apoptosis [54]. Thus, lower TNF*α* level may serve as a mechanism whereby NS398 reduces posthypoxic H9C2 cell apoptosis. However, how does inhibition of COX-2 activity downregulate TNF*α* level and whether TNF*α*-mediated extrinsic apoptosis is affected by NS398 pretreatment are still unclear and warrant further investigation.

Nitric oxide (NO) represents one of the most important defense mechanisms against cell apoptosis, which can either indirectly prevent the caspase-3-like protease activation in cGMP-dependent way or directly inhibit the activation of caspase-3-like protease through S-nitrosylation of the enzyme [55]. Thus, the concomitance of higher intracellular NO level and lower posthypoxic apoptosis in NS398 pretreated cells suggests that inhibition of COX-2 may protect against H/R-induced cell apoptosis through augmentation of NO production. Indeed, the present study proposed two distinct ways to identify how inhibition of COX-2 affects intracellular NO level. (1) ROS/NO balance: ROS are generated intracellularly through multiple metabolic activities. The metabolism of arachidonic acid into specific prostanoids relies on the activity of COX, during which ROS are formed as by-products [5, 56]. The overproduction of ROS was observed in myocardial I/R injury and promoted the development of this ischemic disease [39]. Indeed, in the current study, higher intracellular ROS level was concomitant with induction of COX-2 in H/R-stimulated H9C2 cardiomyocytes, whereas inhibition of COX-2 activity significantly alleviated ROS generation. Of note, elevated formation of ROS closely linked to low NO bioavailability, since excessive ROS production not only disturbs the equilibrium of NOS and NO, but also reacts with NO [which is unstable in oxygenated environment] to form nitrite salt (i.e., peroxynitrite), which exacerbates proapoptotic events [57, 58]. Indeed, previous studies from our group reported that treatment with antioxidants N-Acetylcysteine and allopurinol in the myocardial I/R rat model significantly reduced myocardial ROS formation, produced higher NO content in cardiac tissue, and alleviated infarct size [59, 60]. Taken together, these multiple lines of evidence suggest that inhibition of COX-2 may have protected cell apoptosis through attenuation of ROS generation. (2) Akt/iNOS/NO signaling pathway: activation of Akt promotes the mRNA and protein expression of COX-2 in human endometrial cancer cells [21]. However, in the present study, induction of COX-2 in H/R-stimulated H9C2 cardiomyocytes was accompanied by lower phosphorylation of Akt. In addition, inhibition of COX-2 in H/R-stimulated cells promotes activation of Akt, suggesting that Akt/COX-2 cascade may be stimuli-and cell-specific. It has become evident that activation of PI3K/Akt signaling pathway may protect against cardiomyocytes apoptosis through facilitating the NO production during ischemic disease [40]. The formation of NO is catalyzed by nitric oxide synthase, including neuronal NOS, endothelial NOS, and iNOS [58]. Although several reports suggested a role of iNOS mediated production of NO in cardiac damage [61, 62], there are accumulating evidences showing that iNOS exerts cardioprotective effects in acute and chronic heart disease [63–65]. Indeed, in the present study, higher expression of phosphorylated Akt was concomitant with enhanced expression of iNOS and intracellular NO production in NS398 pretreated H9C2 cardiomyocytes, whereas LY294002 (Akt kinase specific inhibitor) or 1400W (iNOS specific inhibitor) cancelled NS398-mediated cardioprotective effects. Of note, 1400W treatment was associated with lower iNOS protein expression as compared to H/R group. Although the underlying mechanism is still unclear, it could be possible that 1400W may interrupt the transcriptional control of the mRNA expression of iNOS* via* inhibition of inflammatory stress [66, 67]. Taken together, these observations suggested that inhibition of COX-2 may protect cell apoptosis through activation of Akt/iNOS/NO signaling pathway.

Findings of the current study suggest that NF*κ*B is a major player/mediator in H/R-induced expression of COX-2 in H9C2 cardiomyocytes. However, besides the NF*κ*B/COX-2 signaling, NF*κ*B also controls hundreds of gene expressions which are involved in inflammation, cell growth, differentiation, and cell apoptosis [68]. In particular, accumulating evidences have demonstrated that NF*κ*B promotes cell survival due to the upregulation of antiapoptotic genes, including A1/Bfl1 [69], Bcl-X_L_ [70], and Nr13 [71]. Thus, transcriptional factor NF*κ*B exerts both pro- and antiapoptotic effects in the context of apoptotic stimulus [72]. In the present study, when Helenalin was applied to block NF*κ*B activity, COX-2 was sequentially blocked, but other downstream signals of NF*κ*B (like NF*κ*B-mediated anti-apoptotic genes) may also be suppressed, and this could be the reason why Helenalin did not block LDH release as NS398 did.

**Figure 9:**
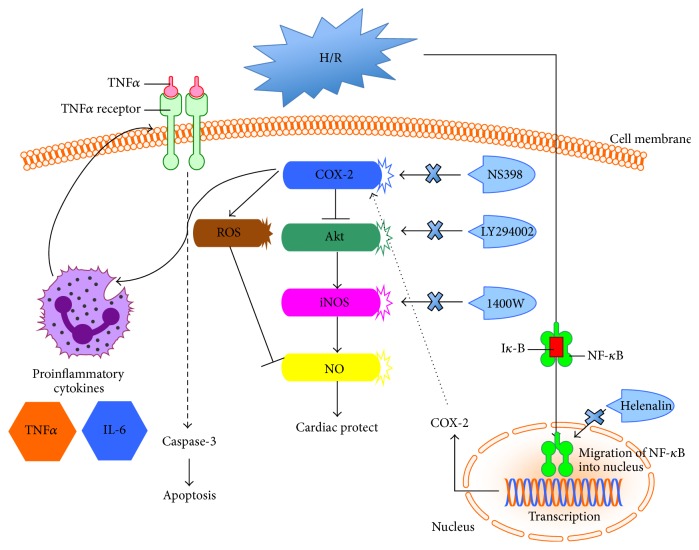
Schematic of proposed signaling involved in the effect of COX-2 in H/R-induced cardiomyocytes apoptosis. In cardiomyocytes, COX-2 was strongly induced in response to H/R stimulation in NF*κ*B-dependent way. Acute pretreatment with NS398, the COX-2 specific inhibitor, can protect against H/R-induced cell injury and apoptosis through attenuation of proinflammatory cytokines expression, ROS formation, and activation of Akt/iNOS/NO signaling pathway.

In the clinical settings, higher incidence of myocardial infarction has been reported in individuals who chronically take Vioxx and Celebrex, specific inhibitors of COX-2 [73, 74]; thus these selective inhibitors of COX-2 are not recommended for long-term use for patients. However, consistent with the work done in experimental animal models [12–15], the present study demonstrated that COX-2 was strongly induced by NF*κ*B activation in response to H/R stimulation. Acute pretreatment with NS398, the COX-2 specific inhibitor, can protect against H/R-induced cell injury and apoptosis through attenuation of proinflammatory cytokines expression, reduction of ROS formation, and activation of Akt/iNOS/NO signaling pathway in cardiomyocytes (Figure 9).

## 5. Conclusion

From a practical standpoint, further understanding on how COX-2 impacts cardiomyocytes apoptosis may have profound clinical implications in the light of the significant role of apoptosis in ischemic heart disease and the widespread use of COX-2 selective inhibitors. In conclusion, findings from the current study provide evidence to suggest that activation of NF*κ*B during cardiomyocyte H/R induces the expression of COX-2 and that higher COX-2 expression during H/R exacerbates cardiomyocyte H/R injury* via* mechanisms that involve cross talk among inflammation, ROS, and Akt/iNOS/NO signaling.

## Figures and Tables

**Table 1:** Primers used in quantitative real-time polymerase chain reactions.

Gene	Forward sequence 5′→3′	Reverse sequence 5′→3′
*β*-actin	AGGCCAACCGTGAAAAGATG	ACCAGAGGCATACAGGGACAA
IL1*β*	TACCTATGTCTTGCCCGTGGA	ATCATCCCACGAGTCACAGAGG
IL6	ACTTCACAAGTCGGAGGCTT	AGTGCATCATCGCTGTTCAT
TNF*α*	TCTCAAAACTCGAGTGACAAGC	GGTTGTCTTTGAGATCCATGC

IL1*β* = interleukin 1*β*; IL6 = interleukin 6; and TNF*α* = tumor necrosis factor *α*.
